# Exploratory pharmacokinetic/pharmacodynamic range of tigecycline for balancing treatment effect and safety in hospital-acquired pneumonia: a retrospective cohort study

**DOI:** 10.3389/fphar.2026.1880928

**Published:** 2026-09-25

**Authors:** Yating Lu, Ruhua Wei, Mingwei Meng, Wen Cao, Zheng Huang, Boren Li, Xiaobu Lan, Kai Mo, Yane Qin

**Affiliations:** 1 Department of Pharmacy, The Fifth Affiliated Hospital of Guangxi Medical University and The First People’s Hospital of Nanning, Nanning, China; 2 Department of Pharmacy, Guangxi International Zhuang Medicine Hospital, Nanning, China; 3 Department of Pharmacy, Guangxi Academy of Medical Sciences and the People’s Hospital of Guangxi Zhuang Autonomous Region, Nanning, China

**Keywords:** tigecycline, therapeutic drug monitoring, hematological toxicity, hospital-acquired pneumonia, clinical treatment effect and safety

## Abstract

**Introduction:**

Tigecycline (TGC) is a core antibiotic for treating hospital-acquired pneumonia (HAP) caused by multidrug-resistant (MDR) bacteria, but the absence of individualized dosing guidance that balances treatment effect and safety frequently results in suboptimal efficacy or coagulation-related adverse events in clinical practice.

**Methods:**

A single-center retrospective study was conducted, enrolling 100 patients with MDR-HAP who were assigned to a standard-dose group or a high-dose group based on clinical decisions. The efficacy and safety of different TGC dosing regimens were compared between the two groups.

**Results:**

The trough concentration (C_min_), intermediate concentration (C_1/2_), and peak concentration (C_max_), the estimated ratio of the area under the concentration-time curve to the 24h of dosing/minimum inhibitory concentration (eAUIC), pharmacokinetic/pharmacodynamic (PK/PD) target attainment rate, clinical response rate and overall favorable outcome rate in the high-dose group were significantly higher than those in the standard group (*p* < 0.05). The incidence of thrombocytopenia was significantly higher in the high-dose group (36.67% vs. 17.14%, *p* = 0.034), whereas the incidence of hypofibrinogenemia was numerically higher but did not reach statistical significance (40.0% vs. 30.0%, *p* = 0.330). No significant between-group differences were observed in microbiological eradication rate, median survival time, 30-day all-cause mortality, coagulation parameters, gastrointestinal reactions, or liver function damage. Binary logistic regression analysis revealed that eAUIC [OR = 1.394 (1.135-1.712), *p* = 0.002] was an influencing predictor associated with clinical treatment effect. The area under the receiver operating characteristic (ROC) curve for eAUIC was 0.813, with a maximum Youden index of 0.557. The corresponding critical value was determined to be 2.915, with a sensitivity of 96.9% and a specificity of 58.8%. Furthermore, eAUIC was independently associated with both hypofibrinogenemia and thrombocytopenia. We identified an exploratory candidate eAUIC range of 2.915 – 3.565 that was associated with both clinical treatment effect and coagulation-related adverse events.

**Discussion:**

This eAUIC range was derived from retrospective data and should be framed explicitly as a hypothesis to be tested, clinical applicability requires confirmation through prospective studies and external validation. Therapeutic drug monitoring may assist in optimizing therapy, but prospective studies are needed to confirm these observations.

## Introduction

Hospital-acquired pneumonia (HAP) and ventilator-associated pneumonia (VAP) are some of the most common and serious infections occurring in hospitalized patients. The mortality rate for HAP ranges from 15.5% to 38.2% and can reach 60% in some specific settings, especially when caused by multidrug-resistant (MDR) strains (Micek et al., 2016).

Tigecycline (TGC), the first glycylcycline of the tetracycline antibiotic class, is approved for the treatment of complicated skin and skin-structure infections (cSSSI) and complicated intra-abdominal infections (cIAI) in China (Meagher et al., 2005). Due to the scarcity of other effective antimicrobials, the use of TGC is frequently extended to the treatment for MDR infections, such as HAP (Zhou et al., 2019). The recommended initial dose of TGC for injection is 100 mg, followed by 50 mg every 12 h. Several studies have reported a high percentage of treatment failure among patients treated with the recommended dose of TGC (Kim et al., 2016; Xie et al., 2014; Shen et al., 2015). A meta-analysis study indicated that higher doses of TGC lead to lower all-cause mortality and higher clinical cure and microbiological eradication rates (Zha et al., 2020). Nowadays, the IDSA guidelines recommend high-dose TGC (initial dose of 200 mg, followed by 100 mg every 12 h) as an alternative option for the treatment of infections caused by carbapenem-resistant Enterobacteriaceae (CRE) and carbapenem-resistant *Acinetobacter baumannii* (CRAB) (Tamma et al., 2022). While a high dose of TGC can achieve better clinical outcomes, it concurrently increases the likelihood of gastrointestinal symptoms, coagulation dysfunction and liver toxicity among critically ill patients (Postier et al., 2004; Ellis-Grosse et al., 2005; Oliva et al., 2005; Lin et al., 2023).

Currently, therapeutic drug monitoring (TDM) is increasingly recognized as an essential tool for individualized therapy. Numerous studies have demonstrated associations between TGC pharmacokinetic (PK) and pharmacodynamic (PD) indices and antimicrobial activity or clinical outcomes (Koomanachai et al., 2009). The ratio of the 24-h area under the concentration–time curve to the minimum inhibitory concentration (AUC_0_–_24_/MIC) is a core PK/PD exposure marker, conventionally termed AUIC. Prior exposure-response analyses for HAP proposed AUIC ≥ 4.5 as the target threshold for optimal tigecycline efficacy (Bhavnani et al., 2012; Xie et al., 2017). Nevertheless, calculating a complete AUC curve demands serial blood sampling at multiple time points, which is difficult to implement in routine clinical care. To resolve this limitation, we adopted a three-point limited sampling strategy in the present cohort and computed an estimated AUIC, abbreviated as eAUIC, via the linear trapezoidal approximation method. This simplified index serves as a practical surrogate for full PK profiling. To date, data linking this estimated eAUIC metric to TGC-related adverse events remain scarce, and the safe upper eAUIC cutoff for balanced efficacy and toxicity has not been well defined. Accordingly, we conducted this real-world retrospective cohort analysis to compare eAUIC levels and corresponding clinical efficacy across different TGC dosing groups, aiming to provide practical references for rational TGC administration and TDM-guided management in hospitalized patients.

## Materials and methods

### Patients

Data were gathered from hospitalized patients who received TGC treatment for infection at a class-A tertiary hospital in Guangxi, spanning from August 2022 to June 2025. The study patients were recruited and assigned to either the standard-dose (SD) or high-dose (HD) groups for the treatment of HAP depending on the doctors’ decisions. The SD patients were treated with TGC 50 mg every 12 h after a 100 mg loading dose. The HD subjects received 100 mg every 12 h after a 200 mg loading dose. The inclusion criteria for this study were as follows: patients must have undergone TGC therapy for a minimum duration of 3 days and had regular monitoring of plasma concentrations throughout the treatment period. All enrolled patients were required to have clinical specimens submitted for culture within 72 h before or after treatment initiation, with positive culture results and definitive pathogen identification by microbiological methods. Conversely, the exclusion criteria encompassed incomplete clinical medical records, patients with pre-existing coagulation dysfunction, individuals aged below 18 years, fibrinogen (FIB) lower than 2.0 g/L, liver failure, those who had received drug combinations affecting coagulation, and those with known allergies to TGC. Ethical approval for this study was granted by the ethics committee of the First People’s Hospital of Nanning (No. LW2025-DECISION-024).

### Sample collection and analysis

Blood samples were collected after at least six doses of TGC (on day 4 of treatment) to achieve steady-state. From each patient, TGC was administered as a 30-min intravenous infusion, three plasma TGC concentrations were recorded. C_min_ (serum concentration at 30 min before the next dose), C_1/2_ (serum concentration at 6 h after dosing), and C_max_ (serum concentration at 30 min after the end of infusion). The plasma sample was centrifuged at 4,000 r/min for 2 min, and then stored in a refrigerator −80 °C before testing. Serum TGC concentrations were analyzed using a validated two-dimensional liquid chromatographic system at the department of pharmacy (FLC-2801 system, Demeter Instrument Co. Ltd., Changsha, China). The first separation system was performed on column ASTON SNX5 (4.6 mm × 50 mm, 5 μm, ANAX, Changsha, Hunan China), and the second separation system was performed on column ASTON SCB-2A (4.6 mm × 250 mm, 5 μm, ANAX, Changsha, Hunan China), and both were performed under a constant temperature of 45 °C. The mobile phase of LC1 was TGCL-1D (Demeter Instrument Co. Ltd., Changsha, China), while mobile Phase of LC2 was TGCL-2B (Demeter Instrument Co. Ltd., Changsha, China). The flow rate was set at 1.2 mL/min, and the UV detector was set at 340 nm. A linear range was found from 45.23 ng/mL to 2,412 ng/mL (r^2^ = 0.9999), with the precision and accuracy are both within 15%.

### Data collection

The demographic and clinical data for the patients, which included age, gender, Weight, height, temperature, and habitual history. Laboratory parameters including blood cell counts, procalcitonin (PCT), C-reactive protein (CRP), serum creatinine (Scr), total bilirubin (TB), albumin, treatment duration and combination therapy were monitored periodically. To evaluate treatment effects, we measured FIB, prothrombin time (PT), activated partial thromboplastin time (APTT), thrombin time (TT), platelet count (PLT), international normalized ratio (INR), alkaline phosphatase (ALP), and alanine aminotransferase (ALT). Renal function was assessed by calculating the creatinine clearance (CrCl) using the Cockcroft-Gault formula. CrCl (mL/min) (for males) = (140 - age) × body weight (kg)/[0.818 × serum creatinine (μmol/L)]; CrCl (for females) = CrCl (male) × 0.85. Consistent with previous studies, renal insufficiency was defined as CrCl <80 mL/min. The estimated area under the concentration-time curve from 0 to 24 h (AUC_0–24_) was calculated using the linear trapezoidal approximation (Xu et al., 2019), with the calculation based on the 12-h AUC (AUC_0-12_) per the formula: AUC_0-24_ = 2 × AUC_0-12_. AUC_0-12_ was calculated as: AUC_0-12_ = [(t_1_ × C_max_) + (t_2_ − t_1_) × (C_max_ + C_1/2_) + (t_0_ − t_2_) × (C_min_ + C_1/2_)]/2, where t_0_ = 30 min before the next dose, corresponding to C_min_; t_1_ = 30 min after the end of infusion, corresponding to C_max_; and t_2_ = 6 h after dosing, corresponding to C_1/2_.

### Efficacy assessment

The clinical treatment effect was evaluated according to the “Technical Guidelines for Antimicrobial Clinical Trials” issued by the former China Food and Drug Administration, assessing three aspects: clinical response, microbiological outcome, and overall outcome (Writing Group of Guidance for Clinical Trials of Anti-Bacterial Drugs, 2014).

Clinical response was categorized into four levels: clinical cure, clinical improvement, clinical failure, and clinical relapse. The specific criteria were as follows:

Clinical cure: Complete resolution of fever (temperature ≤37.2 °C for ≥72 h), normalization of white blood cell count and procalcitonin (or ≥80% reduction from baseline), and complete resolution of infection-related signs and symptoms at the end-of-therapy visit.
Clinical improvement: Partial but clinically meaningful resolution of signs and symptoms, defined as a ≥50% reduction in the total clinical symptom score (based on a predefined scoring system covering fever, local signs of infection, and laboratory inflammatory markers), without need for antibiotic escalation or rescue therapy.
Clinical failure: Persistent or worsening signs/symptoms of infection, need for antibiotic change or escalation, or death directly attributed to the baseline infection (e.g., septic shock, progressive multi-organ failure due to uncontrolled infection, or pneumonia-related respiratory failure).
Clinical relapse: Recurrence of infection after initial improvement, which was classified as clinical failure if occurring at or before the end-of-therapy visit.

For patients who died of causes clearly unrelated to infection (e.g., underlying malignancy progression, acute cardiovascular events, trauma, or other non-infectious causes), clinical response was evaluated based on their infection-related signs and symptoms at the time of death or at the last available assessment prior to death. If the infection had been controlled, they could be classified as clinical cure or clinical improvement; if the infection remained uncontrolled or worsened, they were classified as clinical failure.

Microbiological outcomes were evaluated at both the patient level and the pathogen level for all baseline isolates, with the following categories:

Eradication: The baseline pathogen was not isolated from the original infection site at the end-of-therapy visit.
Presumed eradication: Clinical response was achieved, but follow-up cultures could not be obtained because of clinical improvement (e.g., no sputum production, wound healing, or invasive sampling was no longer ethically or practically feasible). This category was applied only when at least two prior cultures (baseline and one on-treatment) were negative or showed no growth of the baseline pathogen.
Persistence: The baseline pathogen was still isolated from the original infection site at the end-of-therapy visit.
Presumed persistence: Clinical failure occurred, but follow-up cultures were not obtained; this was assumed to represent persistence of the baseline pathogen.
Superinfection: A new pathogen was isolated from the original infection site during or after therapy, accompanied by new or worsening clinical signs/symptoms requiring additional antimicrobial therapy.

The overall outcome was defined as follows:

Overall favorable outcome (success): Clinical response (cure or improvement) achieved and microbiological eradication or presumed eradication.
Overall unfavorable outcome (failure): Clinical response (failure or relapse) or microbiological persistence/presumed persistence/superinfection or both.

For MIC interpretation, recovered isolates were tested by the broth microdilution method following CLSI guidelines. For CRE, FDA breakpoints (≤2 μg/mL susceptible, 4 μg/mL intermediate, ≥8 μg/mL resistant) were applied, as CLSI provides no official breakpoints for this group. For CRAB, given the absence of CLSI/EUCAST/FDA breakpoints, we applied the Chinese expert consensus (≤2 μg/mL susceptible, 4 μg/mL intermediate, ≥8 μg/mL resistant) (Chinese Association for International Exchange of Medical and Healthcare Promotion Clinical Microbiology and Infection Branch et al., 2020).

### Safety assessment

The judgment of adverse drug reactions is based on the Technical Guidelines for Clinical Trials of Antibacterial Drugs. Prior to initiating TGC therapy, confirm that the patient’s liver function is within the normal range. During treatment, if ALT levels rise to three times or more the upper limit of normal or ALP levels reach twice or more the upper limit of normal, the reaction meets the criteria for defining TGC hepatotoxicity (Li et al., 2026). Collect the values of PLT, FIB, PT, INR and APTT before and after TGC treatment. FIB reduction is diagnosed if the post-treatment FIB level decreases by more than 25% compared to the pre-treatment baseline. Hypofibrinogenemia is defined as a FIB level of <2 g/L (Yan et al., 2024). Thrombocytopenia was diagnosed if either of the following two criteria was met: (1) the post-treatment platelet count was less than 100 × 10^9^/L; or (2) the post-treatment platelet count decreased by more than 50% compared with the pre-treatment baseline value (Thiele et al., 2013; Kawasuji et al., 2021).

### Statistical analyses

Data processing was performed using IBM SPSS version 23.0 (SPSS Inc., Chicago, IL). Continuous variables were first tested for normality using the Shapiro–Wilk test, with *p* > 0.05 considered as indicating a normal distribution and *p* ≤ 0.05 indicating a departure from normality. Normally distributed data were expressed as mean ± standard deviation and compared using the *Student’s t-test*; non-normally distributed data were expressed as median and interquartile range (IQR) and compared using the Mann–Whitney U test. Categorical variables were expressed as number (%) and compared using the *Pearson chi-square test* or *Fisher’s exact test*, as appropriate. Binary logistic regression was used to identify influencing factors for clinical efficacy and adverse drug reaction occurrence. Candidate variables were first assessed in univariate logistic regression analyses. All variables with *p* < 0.10 in univariate analysis were entered into multivariate logistic regression models using a forward stepwise (likelihood ratio) approach. The sensitivity and specificity of the eAUIC in the determination of outcomes were determined using receiver operating characteristic (ROC) curve analysis. The optimal cut-off value was determined using the Youden index. The 95% CIs for sensitivity and specificity at the optimal cut-off were calculated using the Wilson score method (binomial proportion confidence interval). To internally validate the stability of the ROC-derived optimal cut-offs, bootstrap resampling with 1,000 iterations was performed using R version 4.6.0 (R Foundation for Statistical Computing, Vienna, Austria). Given the exploratory and hypothesis-generating nature of this study, no adjustment was made for multiple comparisons (e.g., Bonferroni or FDR) across the various tables and models. Such conservative corrections were not applied to avoid inflating type II error rates and obscuring potentially meaningful signals that warrant further investigation. For 30-day survival analysis, in addition to the Kaplan-Meier method with log-rank and Breslow tests, a multivariable Cox proportional hazards regression model was constructed to identify associated factor associated with survival. Hazard ratios (HR) with 95% CIs were reported for all covariates. The C-index was used to evaluate the discriminative ability of the model. A P-value <0.05 was considered statistically significant.

## Results

### Demographics and clinical characteristics

A total of 100 patients who fulfilled the inclusion and exclusion criteria were considered for the retrospective analysis, with 30 and 70 patients assigned to the HD and SD groups, respectively. Baseline comparisons of demographic data, underlying comorbidities, intra-hospital clinical features, ICU admission status, coagulation and liver function indicators, as well as pathogen distribution and MIC levels between the two groups are presented in Table 1. No statistically significant intergroup differences were observed in age, sex, chronic underlying diseases, routine laboratory biomarkers, intra-hospital clinical features, ICU admission proportion, overall positive rates of CRAB and CRE, or the main MIC stratification of isolated strains (all *p* > 0.05).

**TABLE 1:** Bivariate comparisons of clinical characteristics between standard-dose groups and high-dose groups.

Variables	SD group (N = 70)	HD group (N = 30)	P value
Sex (male/female)	48/22	25/5	0.128
Age (year)	76.5 (62.5, 83.75)	68 (59.5, 77.75)	0.051
Baseline indicators			
PT (s)	14.8 (13.5, 16.08)	14.8 (12.35, 15.58)	0.282
APTT (s)	41 (33.5, 44.23)	38.85 (32.58, 46.08)	0.575
INR	1.21 (1.12, 1.37)	1.25 (1.12, 1.39)	0.922
FIB (g/L)	4.68 (3.72, 5.73)	4.1 (3.24, 5.71)	0.128
PLT (×10^9^/L)	255 (170.25, 324.5)	302 (201.75, 374)	0.219
AST (U/L)	29 (21.43, 56.15)	30 (20.6, 39.33)	0.365
ALT (U/L)	23.5 (12.33, 42)	16.25 (10.8, 30.1)	0.222
ALP (U/L)	104 (79, 131.35)	98.85 (73, 139.3)	0.568
DBIL (U/L)	4.6 (3.63, 6.78)	4.25 (2.55, 6.7)	0.152
IBIL (μmol/L)	3.6 (2.3, 5.5)	4.25 (2.38, 5.85)	0.769
WBC (×10^9^/L)	11.19 (8.86, 14.26)	10.99 (8.81, 15.58)	0.685
CRP (mg/L)	60.8 (27.48, 119.15)	70.24 (36.3, 117.36)	0.701
Albumin (g/L)	30.44 ± 4.04	31.44 ± 4.58	0.3
Underlying diseases			
Intracerebral hemorrhage (n %)	7 (10.00)	5 (16.67)	0.546
Cerebral infarction (n %)	11 (15.71)	5 (16.67)	0.905
Chronic obstructive pulmonary disease (n %)	6 (8.57)	4 (13.33)	0.716
Cardiac insufficiency (n %)	7 (10.00)	2 (6.67)	0.879
Renal insufficiency (n %)	30 (42.86)	15 (50.00)	0.511
Hypertension (n %)	28 (40.00)	13 (43.33)	0.756
Diabetes mellitus (n %)	14 (20.00)	4 (13.33)	0.426

Variables	SD group (N = 70)	HD group (N = 30)	P value
Intra-hospital clinical features			
Mechanical ventilation (n %)	43 (61.43)	18 (60)	0.893
Vasoactive agents (n %)	27 (38.57)	17 (56.66)	0.095
Bacteremia (n %)	26 (37.14)	13 (43.33)	0.561
ICU admission (n%)	17 (24.29)	12 (40.00)	0.113
Pathogen			
Co-infection with *Acinetobacter baumannii* and *Klebsiella pneumoniae* (n%)	3 (4.29)	2 (6.67)	-
MIC = 8 μg/mL (n %)	1 (1.43)	0 (0.00)	-
MIC = 2 μg/mL (n %)	2 (2.86)	2 (6.67)	-
*Acinetobacter baumannii* (n %)	38 (54.29)	20 (66.67)	0.250
MIC = 8 μg/mL (n %)	1 (1.43)	1 (3.33)	-
MIC = 4 μg/mL (n %)	0 (0.00)	1 (3.33)	-
MIC = 2 μg/mL (n %)	37 (52.86)	18 (60.00)	0.511
*Klebsiella pneumoniae* (n %)	27 (38.57)	8 (26.67)	0.253
MIC = 4 μg/mL (n %)	1 (1.43)	0 (0.00)	-
MIC = 2 μg/mL (n %)	24 (34.29)	7 (23.33)	0.278
MIC = 1 μg/mL (n %)	2 (2.86)	1 (3.33)	-
*Escherichia coli* (n %)	1 (1.43)	0 (0.00)	-
MIC = 2 μg/mL (n %)	1 (1.43)	0 (0.00)	-
*Enterobacter cloacae* (n %)	1 (1.43)	0 (0.00)	-
MIC = 2 μg/mL (n %)	1 (1.43)	0 (0.00)	-

PT, prothrombin time; APTT, activated partial prothrombin time; INR, international normalized ratio; FIB, fibrinogen; PLT, platelet; AST, aspartate aminotransferase; ALT, alanine transaminase; ALP, alkaline phosphatase; DBIL, direct bilirubin; IBIL, indirect bilirubin; WBC, white blood cell; CRP, C-reactive protein.

P values for categorical variables were determined by Fisher’s exact test. Subgroups with a cell count less than five were only presented descriptively without intergroup comparison and marked as “-”.

In terms of pathogenic distribution, CRAB was the predominant isolated pathogen, accounting for 58% of all patients, followed by CRE at 35%. A total of 5% of patients presented co-infection with both CRAB and CRE. Regarding MIC distribution, the majority of CRAB strains and CRE strains exhibited an MIC of 2 μg/mL. Additionally, CRAB strains with MIC values of 8 μg/mL and 4 μg/mL were also identified, while CRE strains with MIC values of 4 μg/mL and 1 μg/mL were documented. Rare pathogens including *Escherichia coli* and *Enterobacter cloacae* were only detected in a small number of SD group patients. Detailed categorical data with positive case numbers and constituent ratios of all variables are summarized in Table 1.

### Efficacy analysis

As shown in Table 2, the C_min_, C_max_, C_1/2_, AUC_0-24_, eAUIC, target attainment rate, clinical response rate and overall favorable outcome rate in the HD group were higher than in the SD group (*p* < 0.05). Furthermore, there were no differences in microbiological eradication rate and 30-day all-cause mortality rate between the two groups (*p* > 0.05). The median survival time was 22.5 days (IQR: 15.5–30) in the SD group and 24 days (IQR: 13–30) in the HD group, with no significant difference detected by either the log-rank test (*p* = 0.212) or the Breslow test (*p* = 0.344) (Figure 1; Table 3).

**TABLE 2:** Comparison of the pharmacokinetic parameters and efficacy between the two groups.

Variables	SD group (N = 70)	HD group (N = 30)	P value
C_min_ (ng/mL)	130.99 (88.67, 188.12)	326.11 (232.54, 432.53)	<0.001
C_max_ (ng/mL)	637.8 (428.82, 868.17)	1, 117.25 (666.99, 2053.53)	<0.001
C_1/2_ (ng/mL)	217.02 (169.51, 350.39)	576.95 (396.5, 771.81)	<0.001
AUC_0-24_	5.68 (3.73, 7.03)	10.64 (8.00, 14.56)	<0.001
eAUIC	2.84 (1.85, 3.51)	5.2 (4.17, 7.03)	<0.001
Duration of treatment (days)	13 (10, 16.75)	10 (6.25, 13)	0.007
Target attainment rate (n %)	7 (10.00)	22 (73.33)	<0.001
Microbiological eradication rate (n %)	38 (54.29)	16 (53.33)	0.93
Clinical response rate (n %)	22 (31.43)	18 (60.00)	0.008
Overall favorable outcome rate (n %)	18 (25.71)	14 (46.67)	0.04
All-cause mortality rates (n %)	21 (30.00)	5 (16.67)	0.164

C_min_, Trough concentration; C_max_, Peak concentration; C_1/2_, Concentration at the 6 h after the dosing; AUC_0-24_, area under the concentration-time curve from 0 to 24 h; eAUIC, the estimated ratio of the 24 h area under the concentration-time curve (AUC_0-24_) to the minimum inhibitory concentration (MIC).

**FIGURE 1:**
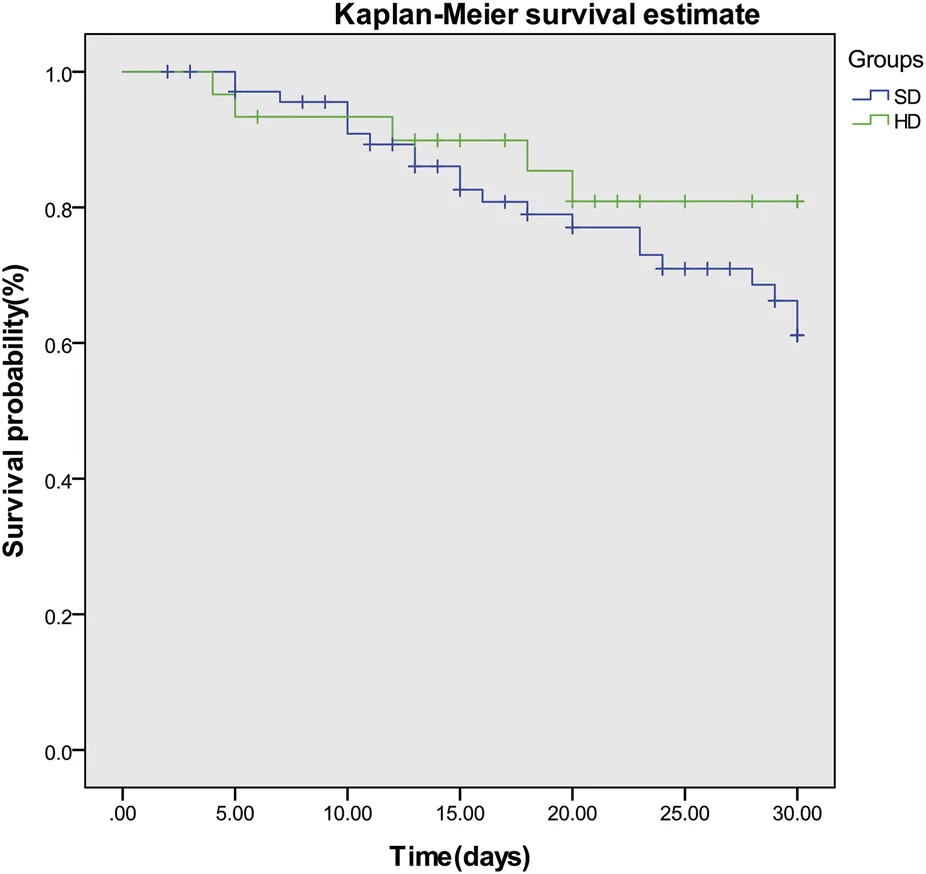
Cumulative survival time of the two groups of patients.

**TABLE 3:** The median survival time of the two groups of patients.

Group	Total patients (N)	Survivors N (%)	Survival times (days)	Log rank (Mantel-Cox)	Breslow
SD group	70	49 (70.00)	22.5 (15.5, 30)	0.212	0.344
HD group	30	25 (83.33)	24 (13, 30)		

To further identify factors associated with 30-day survival, we performed a multivariable Cox proportional hazards regression analysis incorporating tigecycline dose group, renal insufficiency, albumin, eAUIC, age, mechanical ventilation, vasoactive agents, and bacteremia as covariates. As shown in Table 4, renal insufficiency was independently associated with increased mortality risk (HR = 5.001, 95% CI: 1.935–12.927, *p* = 0.001). TGC dose group (high-dose vs. standard-dose) was not significantly associated with 30-day survival (HR = 0.904, 95% CI: 0.280–2.919, *p* = 0.866), consistent with the non-significant log-rank and Breslow test results.

**TABLE 4:** Multivariable Cox regression analysis for 30-day survival (n = 100).

Variables	β	SE	Wald	HR	95%CI	P
eAUIC	−0.183	0.131	−1.391	0.833	0.644–1.078	0.164
Age (per 1-year increase)	0.031	0.016	1.985	1.031	1.000–1.063	0.047
Albumin (per 1 g/L increase)	−0.093	0.052	−1.804	0.911	0.823–1.008	0.071
Renal insufficiency (yes vs. no)	1.610	0.485	3.322	5.001	1.935–12.927	0.001
Mechanical ventilation (yes vs. no)	1.052	0.595	1.768	2.862	0.892–9.182	0.077
Vasoactive agents (yes vs. no)	0.097	0.452	0.215	1.102	0.455–2.671	0.830
Bacteremia (yes vs. no)	−0.404	0.436	−0.927	0.667	0.284–1.569	0.354
High-dose (yes vs. no)	−0.101	0.598	−0.168	0.904	0.280–2.919	0.866

Model performance: Likelihood ratio χ^2^ = 28.134, df = 8, *p* < 0.001; C-index = 0.606. Proportional hazards assumption was satisfied for all covariates (all *p* > 0.05 by Schoenfeld residual test); no significant multicollinearity was detected (all VIF <10, all tolerance >0.1).

The univariate logistic regression analysis showed that the C_min_, C_max_, C_1/2_, and eAUIC were significantly correlated with the efficacy of TGC. The results of a binary logistic regression analysis further identified eAUIC as a predictive biomarker for further evaluating clinical efficacy, with an odds ratio of 1.394 (95% CI: 1.135–1.712, *p* = 0.002) (Table 5). ROC curve analysis was conducted to assess the predictive performance of eAUIC (Figure 2). Upon obtaining the ROC curve, the area for eAUIC under the ROC curves was 0.813. When the optimal eAUIC cut-off value was set at 2.915, the maximum Youden index reached 0.557, with a corresponding sensitivity of 96.9% (95% CI: 83.8%–99.9%) and specificity of 58.8% (95% CI: 46.7%–70.0%).

**TABLE 5:** Univariate and multivariate analysis of factors associated with clinical efficacy in 100 patients with HAP.

Variables	Clinical efficacy		Univariate analysis	Multivariate analysis		
​	Invalid (N = 68)	Effective (N = 32)	P	OR	95%CI	P
C_min_ (ng/mL)	150.1 (90.13, 264.1)	231.35 (140.88, 373.84)	0.011	​	​	​
C_max_ (ng/mL)	621.77 (419.7, 848.25)	1, 029.45 (693.46, 1449.9)	<0.001	​	​	​
C_1/2_ (ng/mL)	226.51 (178.76, 408.75)	507.41 (294.54, 759.55)	<0.001	​	​	​
eAUIC	2.72 (1.82, 3.77)	4.78 (3.34, 6.25)	<0.001	1.394	1.135–1.712	0.002
Albumin (g/L)	31.60 ± 4.60	30.17 ± 3.94	0.133	0.938	0.828–1.062	0.312
Duration of treatment (days)	12 (8, 16)	12.5 (10, 16)	0.821	1.011	0.924–1.107	0.805
Age (year)	73 (59.5, 81.75)	76 (61.75, 83.5)	0.773	0.997	0.964–1.032	0.877
Weight (kg)	60 (50, 66.75)	60 [48.5, 65)	0.970	1.011	0.944–1.083	0.748
Sex (female, n%)	19 (27.94)	8 (25.00)	0.757	1.368	0.298–6.278	0.687
Renal insufficiency (n %)	33 (48.53)	12 (37.5)	0.301	0.499	0.163–1.525	0.223
Combination with tigecycline						
Carbapenems (n %)	27 (39.7)	11 (34.37)	0.608	1.007	0.327–3.106	0.99
Piperacillin tazobactam (n%)	10 (14.7)	2 (6.25)	0.377	0.179	0.017–1.861	0.15
Cefoperazone sulbactam (n %)	17 (25.00)	11 (34.37)	0.33	1.374	0.408–4.62	0.608
Mechanical ventilation (n %)	43 (63.23)	18 (56.25)	0.504	0.414	0.13–1.323	0.137
Vasoactive agents (n %)	28 (41.18)	16 (50.00)	0.407	1.46	0.483–4.409	0.502
Bacteremia (n %)	24 (35.29)	15 (46.87)	0.268	1.709	0.605–4.829	0.312

C_min_, Trough concentration; C_max_, Peak concentration; C_1/2_, Concentration at the 6 h after the dosing; eAUIC, the estimated ratio of the 24 h area under the concentration-time curve (AUC_0-24_) to the minimum inhibitory concentration (MIC).

**FIGURE 2:**
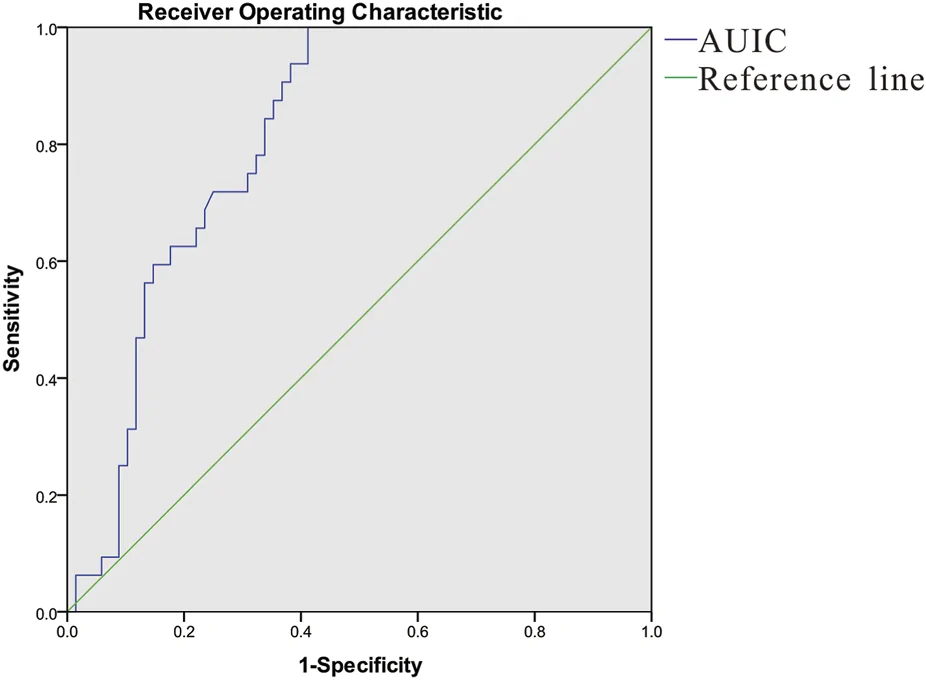
Receiver operating characteristic curve of predictive level of characteristics for clinical efficacy.

### Safety analysis

As summarized in Table 6, we recorded multiple types of TGC-related adverse events across all enrolled patients, with overlapping cases experiencing more than one adverse event simultaneously. The total frequency of each adverse event was as follows: hypofibrinogenemia (33 cases, 33.0%), thrombocytopenia (23 cases, 23.0%), gastrointestinal symptoms (11 cases, 11.0%), and liver dysfunction (7 cases, 7.0%). The incidence of thrombocytopenia was significantly higher in the HD group compared with the SD group (*p* < 0.05). The incidence of hypofibrinogenemia was numerically higher in the HD group, but the difference did not reach statistical significance (*p* = 0.330). No significant between-group differences were observed in PT, APTT, INR, gastrointestinal symptoms, or liver dysfunction (all *p* > 0.05).

**TABLE 6:** Comparison of adverse events profile of TGC in the SD groups and HD groups.

Variables	SD group (N = 70)	HD group (N = 30)	P value
PT (s)	15.95 (14.51, 18.07)	15.1 (13.8, 16.23)	0.138
APTT (s)	46.15 (39.85, 53.52)	44.7 (39.88, 54.58)	0.721
INR	1.30 (1.15, 1.58)	1.29 (1.17, 1.44)	0.537
Hypofibrinogenemia (n %)	21 (30)	12 (40)	0.33
Thrombocytopenia (n %)	12 (17.14)	11 (36.67)	0.034
Gastrointestinal symptoms (n %)	5 (7.14)	6 (20)	0.125
Liver dysfunction (n %)	3 (4.29)	4 (13.33)	0.231

PT, prothrombin time; APTT, activated partial prothrombin time; INR, international normalized ratio.

Binary logistic regression analysis revealed that eAUIC [OR = 1.351 (1.013–1.803),* p* = 0.041] was associated with hypofibrinogenemia. Additionally, eAUIC [OR = 2.075 (1.402–3.071), *p* < 0.001] and the presence of renal insufficiency [OR = 7.585 (1.502–38.301), *p* = 0.014] were significant predictors of thrombocytopenia (Tables 7 and 8). ROC curves of eAUIC were plotted to obtain the cut-off values for hypofibrinogenemia (Figure 3) and thrombocytopenia (Figure 4). Notably, both safety endpoints yielded the same optimal eAUIC cut-off value of 3.565. For hypofibrinogenemia, an eAUIC of 3.565 corresponded to the areas under the ROC curve with 0.676 (95% CI: 0.566–0.786), the maximum Youden index reached 0.323, with a sensitivity of 63.6% (95% CI: 45.1%–79.6%) and specificity of 68.7% (95% CI: 56.2%–79.4%). For thrombocytopenia, an eAUIC of 3.565 corresponded to the areas under the ROC curve with 0.876 (95% CI: 0.802–0.950), which yielded a maximum Youden index of 0.645, with maximum sensitivity and specificity values of 95.7% (95% CI: 78.1%–99.9%) and 68.8% (95% CI: 57.4%–78.7%), respectively. As shown in Figure 5, we further performed bootstrap internal validation with 1,000 resampling iterations to assess the stability of the hypofibrinogenemia predictive threshold. The bootstrap-corrected area under the receiver operating characteristic curve remained 0.676 (95% CI: 0.567–0.777), and the adjusted optimal cut-off slightly shifted to 3.59. At this corrected threshold, the sensitivity was 63.6% (95% CI: 41.37%–97.06%), specificity 68.7% (95% CI: 29.41%–89.2%), and Youden index 0.323 (95% CI: 0.206–0.542).

**TABLE 7:** Univariate and Multivariate analysis of tigecycline-induced hypofibrinogenemia.

Variables	Hypofibrinogenemia		Univariate analysis	Multivariate analysis		
​	No (N = 67)	Yes (N = 33)	P	OR	95%CI	P
C_min_ (ng/mL)	150.08 (89.01, 286.66)	206.11 (148.85, 325.82)	0.027	0.997	0.993–1.001	0.114
C_max_ (ng/mL)	680.9 (437.03, 889.47)	848.42 (585.91, 1395.94)	0.019	​	​	​
C_1/2_ (ng/mL)	239.01 (173.1, 439.96)	409.18 (220.86, 695.8)	0.019	​	​	​
eAUIC	2.95 (1.86, 4.01)	4.31 (2.91, 6.19)	0.004	1.351	1.013–1.803	0.041
Albumin (g/L)	31.54 ± 4.84	30.32 ± 3.36	0.145	0.967	0.865–1.081	0.555
Duration of treatment (days)	12 (7.25, 16)	13 (11, 16.5)	0.192	1.071	0.975–1.177	0.151
Age (year)	73 (58.5, 82)	72 (66, 81.5)	0.342	1.017	0.983–1.051	0.335
Weight (kg)	61 (51.5, 67)	57 (47.75, 62)	0.055	0.949	0.885–1.017	0.14
Sex (female, n%)	18 (26.87)	9 (27.27)	0.966	1.649	0.398–6.835	0.49
Renal insufficiency (n %)	27 (40.3)	18 (54.55)	0.178	1.895	0.685–5.239	0.218
Combination with tigecycline						
Carbapenems (n %)	26 (38.81)	12 (36.36)	0.813	​	​	​
Piperacillin tazobactam (n %)	8 (11.94)	4 (12.12)	1.000	​	​	​
Cefoperazone sulbactam (n %)	15 (22.39)	13 (39.39)	0.075	1.991	0.718–5.523	0.186
Vasoactive agents (n %)	27 (40.3)	17 (51.52)	0.288	1.399	0.528–3.703	0.499
Bacteremia (n %)	24 (35.82)	15 (45.45)	0.353	1.026	0.376–2.802	0.959

C_min_, Trough concentration; eAUIC, the estimated ratio of the 24 h area under the concentration-time curve (AUC_0-24_) to the minimum inhibitory concentration (MIC).

**TABLE 8:** Univariate and Multivariate analysis of tigecycline-induced thrombocytopenia.

Variables	Thrombocytopenia		Univariate analysis	Multivariate analysis		
​	No (N = 77)	Yes (N = 23)	P	OR	95%CI	P
C_min_ (ng/mL)	150.08 (91.44, 244.16)	295.82 (166.1, 356.17)	0.001	​	​	​
C_max_ (ng/mL)	662.36 (490.74, 965.95)	1, 009.84 (657.94, 1278.27)	0.012	​	​	​
C_1/2_ (ng/mL)	239.01 (180.41, 439.96)	560.2 (366.02, 852.58)	0.001	​	​	​
eAUIC	2.96 (1.93, 4.23)	4.31 (3.11, 6.3)	0.003	2.075	1.402–3.071	<0.001
Albumin (g/L)	31.34 ± 4.48	30.49 ± 4.29	0.426	1.13	0.962–1.328	0.137
Duration of treatment (days)	13 (8, 16)	11 (8.25, 16)	0.563	1.053	0.92–1.206	0.452
Age (year)	73 (59, 82)	75 (67.5, 81.5)	0.536	1.027	0.972–1.085	0.343
Weight (kg)	60 (50, 67)	55 (51.5, 62.5)	0.139	0.973	0.874–1.082	0.609
Sex (female, n%)	20 (25.97)	7 (30.43)	0.672	2.22	0.272–18.127	0.457
Renal insufficiency (n %)	27 (35.06)	18 (78.26)	<0.001	7.585	1.502–38.301	0.014
Combination with tigecycline						
Carbapenems (n %)	28 (36.36)	10 (43.48)	0.537	​	​	​
Piperacillin tazobactam (n %)	9 (11.69)	3 (13.04)	1.000	​	​	​
Cefoperazone sulbactam (n %)	20 (25.97)	8 (34.78)	0.409	2.638	0.551–12.625	0.225
Vasoactive agents (n %)	30 (38.96)	14 (60.87)	0.063	1.805	0.422–7.729	0.426
Bacteremia (n %)	25 (32.47)	14 (60.87)	0.014	1.794	0.438–7.346	0.417

C_min_, Trough concentration; eAUIC, the estimated ratio of the 24 h area under the concentration-time curve (AUC_0-24_) to the minimum inhibitory concentration (MIC).

**FIGURE 3:**
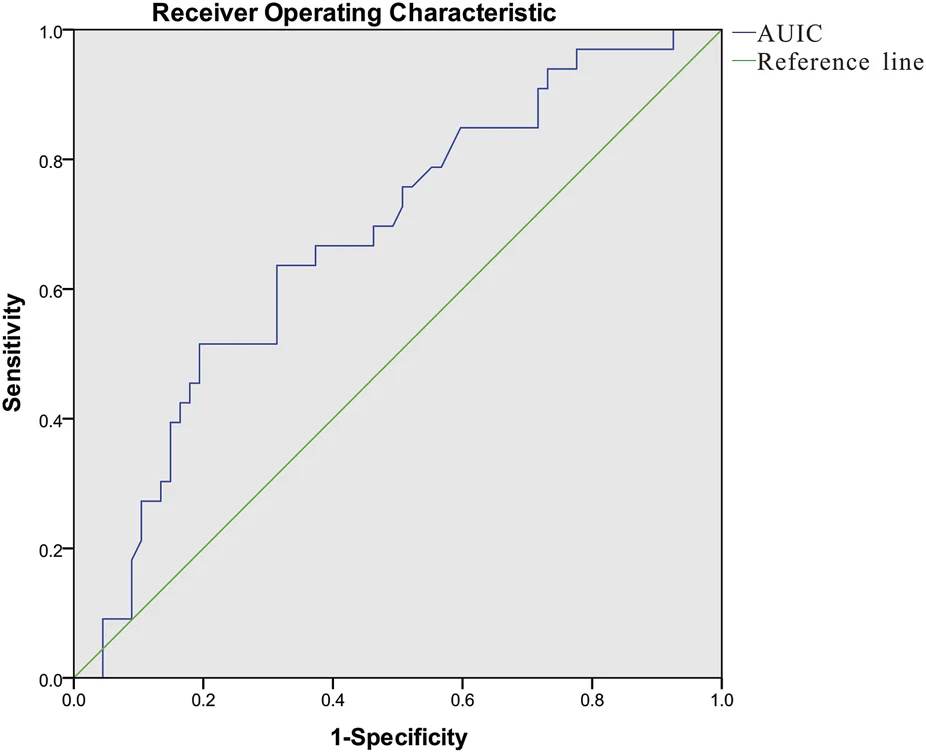
Receiver operating characteristic curve of eAUIC in predicting TGC-induced hypofibrinogenemia.

**FIGURE 4:**
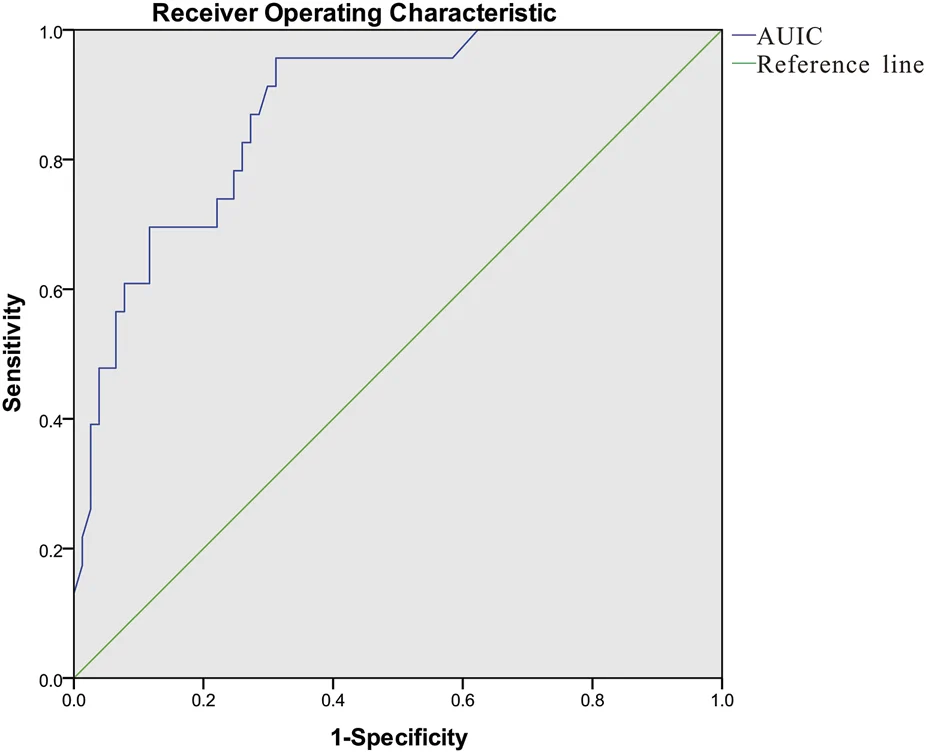
Receiver operating characteristic curve of eAUIC in predicting TGC-induced thrombocytopenia.

**FIGURE 5:**
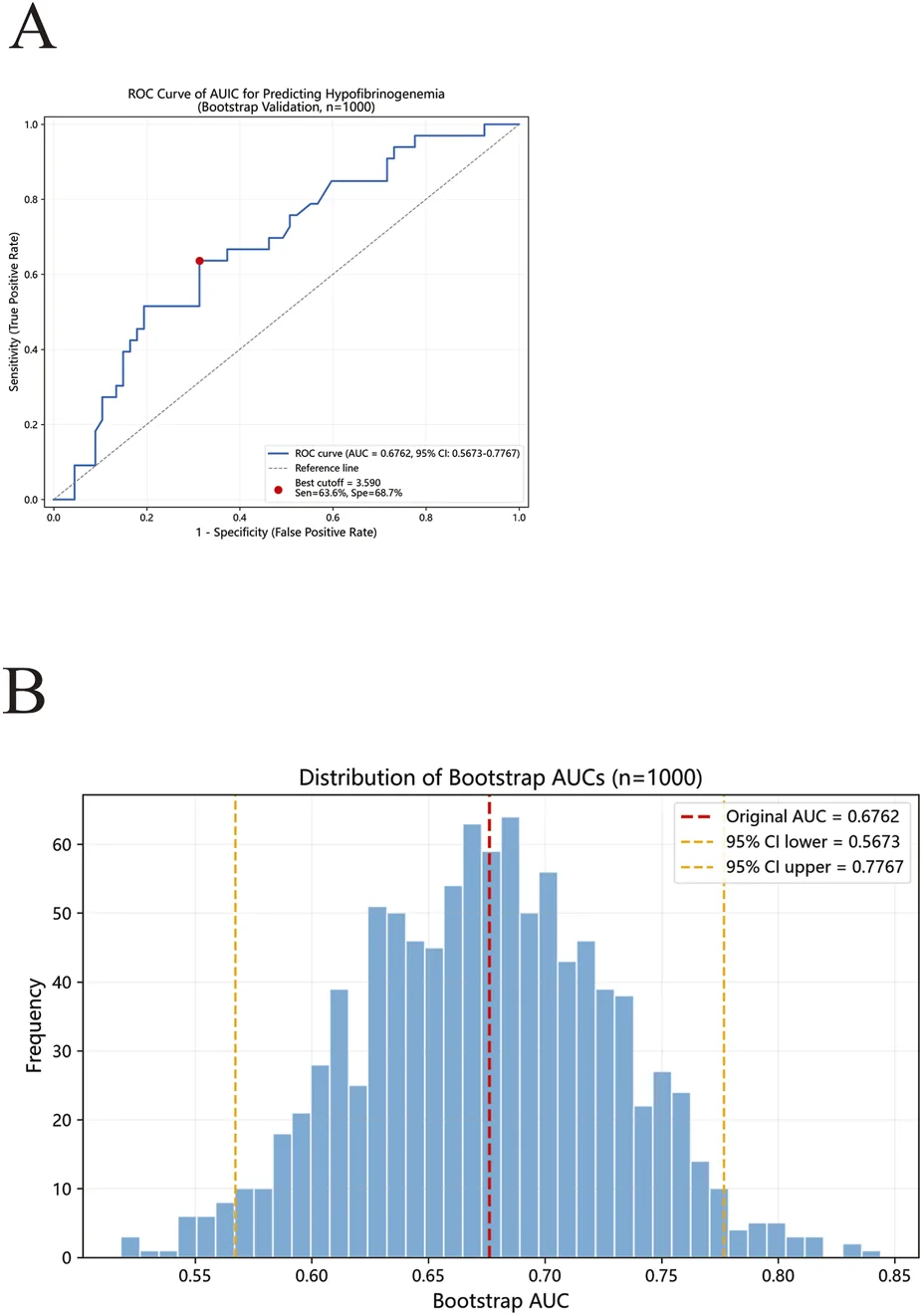
**(A)** Receiver operating characteristic curve of eAUIC for predicting hypofibrinogenemia (Bootstrap validation, n = 1,000). **(B)** Distribution of bootstrap AUCs (n = 1,000).

## Discussion

In China, infections caused by Gram-negative bacteria exhibit a high rate of HAP. According to the China Bacterial Resistance Monitoring Network (CHINET), not only have CRAB and *Klebsiella pneumoniae* retained top-three isolation rates for consecutive years, but their resistance to meropenem has also shown a steady upward trajectory (Hu et al., 2019; Hu et al., 2016). In this study, pathogen detection revealed that 58% of patients were infected with CRAB, while 35% had infections caused by CRE. These findings are largely consistent with the current epidemiological distribution and antimicrobial resistance trends of pathogenic bacteria associated with HAP. The European Society of Clinical Microbiology and Infectious Diseases (ESCMID) guidelines identify TGC as a key therapeutic drug for CRAB and CRE infections (Paul et al., 2022). Notably, the majority of isolates in our cohort exhibited MIC values at the borderline susceptible level (2 μg/mL), which represents the upper limit of the susceptibility breakpoint. Patients infected with such borderline susceptible strains may face considerable therapeutic challenges, as higher drug exposure is required to achieve the pharmacodynamic target. This underscores the clinical importance of PK/PD optimization and TDM-guided dosing when treating HAP caused by these pathogens, particularly in critically ill patients with altered pharmacokinetics.

TGC is characterized as an exposure-dependent antibiotic, and AUIC has been widely recognized as the critical PK/PD parameter for guiding its clinical use and predicting therapeutic outcomes (Craig, 1998). In this retrospective cohort, high-dose TGC showed superior PK/PD target attainment (73.33% vs. 10%, *p* < 0.001), higher clinical response rates (60.00% vs. 31.43%, *p* = 0.008), and higher overall favorable outcome rates (46.67% vs. 25.71%, *p* = 0.04) compared with the standard-dose group. These findings support the clinical rationale for individualized dose optimization, particularly when treating infections caused by pathogens with elevated MICs, but prospective validation is required to confirm this association.

Binary logistic regression revealed that eAUIC was a meaningful biomarker of clinical efficacy. Subsequent ROC curve analyses further validated the predictive capacity of this estimated metric for treatment response. The optimal cut-off value for the eAUIC was determined to be 2.915. It is important to note that this threshold was derived from a sparse-sampling estimation method reflecting total drug exposure, whereas the threshold reported in the prior Phase III trial (Bhavnani et al., 2012) was derived from the full 24-h steady-state area under the curve of the unbound fraction. Given these substantial methodological differences—in both exposure calculation (full AUIC from population PK modeling vs. sparse-point estimation) and protein-binding correction (free vs. total drug), the numerical disparity between the two thresholds does not indicate pharmacological inconsistency but rather highlights the need for caution when comparing PK/PD cut-offs derived from different estimation approaches.

In the multivariable Cox regression analysis for 30-day survival, renal insufficiency emerged as the strongest associated factor of mortality. The magnitude of this effect (5-fold increase in mortality risk) underscores the importance of early identification and intensive management of renal impairment in this patient population. Notably, eAUIC was not significantly associated with 30-day survival in this model. This is consistent with the non-significant log-rank test results between dose groups. The survival benefits brought about by TGC exposure still require a larger sample size to be clearly detected.

While two meta-analyses (Zha et al., 2020; Gong et al., 2019) reported that HD group improves clinical cure and microbial eradication, our data did not show significant differences between HD and SD groups in terms of microbiological eradication rate, 30-day all-cause mortality rate, and median survival time (all *p* > 0.05). Nevertheless, HD group tended to have a lower 30-day all-cause mortality rate (16.67% vs. 30%). Beyond the between-group comparisons, we observed a clear dissociation between clinical and microbiological outcomes: microbiological eradication rates were comparable between HD and SD groups (54.29% vs. 53.33%, *p* = 0.93), while clinical response rates were markedly higher in the HD group (60.00% vs. 31.43%, *p* = 0.008). This inconsistency can be explained by two factors. First, higher TGC exposure in the HD group may have facilitated earlier control of inflammatory symptoms before complete bacterial eradication, consistent with PK/PD principles that clinical improvement can precede microbiological clearance. Second, the shorter treatment duration in the HD group (median 10 vs. 13 days, *p* = 0.007) led to earlier hospital discharge; some patients may have achieved bacterial clearance after the fixed end-of-therapy assessment window, which could not be captured by single-point microbiological testing. In addition, two confounding factors may further widen this discrepancy: the difficulty in distinguishing true infection from bacterial colonization during specimen culture, and multiple non-infectious comorbidities that may have masked the mortality benefit of high-dose TGC.

Prior real-world studies (S et al., 2021; Wang et al., 2022; Campany-Herrero et al., 2020; Fa et al., 2020) have reported that the adverse drug reactions of TGC are dominated by gastrointestinal reactions (diarrhea, nausea), coagulation disorders, thrombocytopenia, and hepatic impairment, which is consistent with the situation we observed. In our study, hypofibrinogenemia and thrombocytopenia were particularly prevalent. The incidence of thrombocytopenia was significantly higher in the HD group compared with the SD group (36.67% vs. 17.14%, *p* = 0.034). The incidence of hypofibrinogenemia was numerically higher in the HD group (40.0% vs. 30.0%), but the difference did not reach statistical significance (*p* = 0.330). No statistically significant differences were observed between the two groups in other coagulation parameters (PT, APTT and INR) (all *p* > 0.05). The differential patterns observed between these two hematological adverse events suggest that they may involve distinct pathophysiological pathways, which remain to be elucidated. In this context, routine laboratory monitoring is essential for early detection of coagulation-related adverse events, eAUIC assessment and laboratory monitoring serve complementary rather than mutually exclusive roles. The former provides an integrated measure of systemic drug exposure that informs dose optimization, whereas the latter enables real-time detection of emerging toxicities. The parallel use of both approaches may offer a more comprehensive strategy for optimizing the benefit–risk balance in patients receiving TGC therapy.

Although combination with cefoperazone-sulbactam did not reach statistical significance as a risk factor in our binary logistic regression analysis (possibly due to limited sample size), a large cohort study (Wang et al., 2020) reported coagulation abnormalities in 9.2% and bleeding events in 4.2% of cefoperazone-sulbactam users. Therefore, while our statistical findings do not support a deterministic warning, we suggest that clinicians remain vigilant regarding FIB and coagulation function when this combination is used, particularly in patients with additional risk factors.

Binary logistic regression analysis revealed that eAUIC was an associated factor for both hypofibrinogenemia (OR = 1.351, *p* = 0.041) and thrombocytopenia (OR = 2.075, *p* < 0.001), while concurrent renal insufficiency (OR = 7.585, *p* = 0.014) further increased the risk of thrombocytopenia. Clinicians must recognize the need to balance efficacy and safety in clinical practice. While the precise relationship between renal insufficiency and thrombocytopenia requires further clarification, we consider it clinically prudent to strengthen hematologic monitoring in this population. We recommend that clinicians maintain a high index of suspicion for thrombocytopenia in patients with renal insufficiency receiving tigecycline. Future prospective studies collecting standardized illness severity data are needed to disentangle confounding factors and clarify the mechanisms linking renal function, tigecycline exposure, and hematological toxicity.

ROC curve analysis identified two distinct eAUIC cut-off values: 2.915 for favorable clinical response and 3.565 for both hypofibrinogenemia and thrombocytopenia. Notably, both coagulation-related safety endpoints shared the same optimal threshold, suggesting that monitoring for hematological toxicity can be guided by a unified eAUIC ceiling. To explore the balance between efficacy and safety, we propose a candidate eAUIC range (2.915–3.565) assembled from two separate ROC cut-offs. However, eAUIC attainment within this range should be interpreted as an exploratory biomarker rather than a therapeutic target, as some patients may still respond favorably even when eAUIC falls below 2.915, and toxicity may occur even when eAUIC is below 3.565. External validation is required before this range can be considered for clinical application.

## Limitations

This study had several limitations that warrant discussion. First, the sample size of this single-center retrospective cohort was relatively small (n = 100), with limited positive events for each binary endpoint. The events-per-variable (EPV) ratios of multivariate logistic regression models ranged from 2.3 to 4.0, which is below the standard EPV ≥10 threshold for confirmatory research. Moreover, we did not apply formal multiple comparison correction for all statistical tests to prevent masking the core PK/PD signals of eAUIC. As such, all findings are exploratory and may not be generalizable to other clinical settings. Second, we lacked critical HAP-related clinical assessment tools [Sequential Organ Failure Assessment (SOFA) scores, Acute Physiology and Chronic Health Evaluation II (APACHE II), and Clinical Pulmonary Infection Score (CPIS)] that quantify disease severity and infection status, the absence of these data may have confounded our ability to accurately associate TGC dosing with efficacy and safety. Third, without long-term follow-up data, we cannot comment on whether TGC use affects patients’ long-term outcomes beyond the study period. Fourth, the AUC_0-24_ values were estimated using a three-point linear trapezoidal method rather than full pharmacokinetic profiling. While we acknowledge that population pharmacokinetic (PopPK) modeling combined with Bayesian forecasting is the gold standard, extensive blood sampling was not feasible in this population. To reduce the bias inherent in the conventional two-point peak-trough approach, we intentionally included a mid-interval sampling point (6 h post-dose) to better capture the two-compartment distribution phase of TGC. This pragmatic limited-sampling approach is supported by previous systematic reviews demonstrating that properly designed sparse-sampling strategies can provide acceptable AUC_0–24h_ estimates in clinically constrained settings (Sprague and Ensom, 2009). Future research should combine the PopPK-Bayesian modeling to further verify and refine the findings of this study. Fifth, the proposed exploratory eAUIC range was derived and tested within the same single-center retrospective cohort without external validation. Therefore, these findings should be considered hypothesis-generating, and prospective multi-center studies with larger sample sizes are needed to validate the clinical applicability of this range.

## Conclusion

In conclusion, this exploratory study identifies eAUIC as a potential integrated exposure index that may assist in balancing treatment effect and hematological safety in TGC-treated HAP patients. However, it is important to emphasize that eAUIC remains an exploratory, hypothesis-generating metric and has not yet been validated as a routine clinical target for dose individualization. Our findings suggest that combining TDM, eAUIC assessment, and routine laboratory monitoring of coagulation parameters (including FIB and PLT) may offer a more comprehensive strategy for optimizing TGC use in MDR-related HAP, though this integrative approach requires prospective evaluation.

Based on our preliminary data, the exploratory candidate eAUIC range of 2.915–3.565 was associated with a favorable balance between treatment response and hematological toxicity in this single-center cohort, with the risks of hypofibrinogenemia and thrombocytopenia rising notably when eAUIC exceeded 3.565. Nevertheless, this range was derived from ROC analyses with bootstrap internal validation in a relatively small sample and requires external validation in independent populations before any clinical application can be recommended. For patients with renal insufficiency, heightened vigilance for thrombocytopenia and regular PLT monitoring appear warranted, and TDM may be considered to assess drug exposure; however, current data do not support routine dose modification based solely on renal function. Regular monitoring of coagulation function (particularly FIB) and PLT counts during TGC therapy, especially in patients receiving high-dose regimens remains prudent.

Future research should focus on multi-center, large-scale prospective studies to develop a regional PopPK-Bayesian model for TGC in HAP patients, which would further validate optimal dosing regimens across pathogens with different MIC values and provide more robust evidence for individualized therapy.

## Data Availability

The original contributions presented in the study are included in the article/supplementary material, further inquiries can be directed to the corresponding authors.
